# Displacement and stress distribution of the craniomaxillofacial complex under different surgical conditions: a three-dimensional finite element analysis of fracture mechanics

**DOI:** 10.1186/s12903-021-01941-1

**Published:** 2021-11-22

**Authors:** Junjie Chen, Yuhan Xu, Chengri Li, Lingling Zhang, Fang Yi, Yanqin Lu

**Affiliations:** Hunan Key Laboratory of Oral Health Research & Hunan 3D Printing Engineering Research Center of Oral Care & Hunan Clinical Research Center of Oral Major Diseases and Oral Health & Academician Workstation for Oral-maxilofacial and Regenerative Medicine & Xiangya Stomatological Hospital & Xiangya School of Stomatology, Central South University, Hunan 410008 Changsha, China

**Keywords:** Maxillary transverse deficiency, Osteotomy-assisted arch expansion, Finite element analysis, Palatal suture, Cone-beam computed tomography

## Abstract

**Objective:**

To provide a simplified treatment strategy for patients with maxillary transverse deficiency. We investigated and compared the fracture mechanics and stress distribution of a midline palatal suture under dynamic loads during surgically-assisted rapid palatal expansion.

**Methods:**

Based on the cone-beam computed tomography (CBCT) data of a 21-year-old female volunteer, a three-dimensional model of the cranio-maxillofacial complex (including the palatal suture) was constructed. A finite element analysis model was constructed based on meshwork. After the yield strength of the palatal suture was set, an increasing expansion force (0–500 N) was applied within 140 ms to calculate the time–load curve, which mimicked nonsurgical bone expansion (model A). The same method was used to evaluate the fracture process, time and stress distribution of the palatal suture in maxillary lateral osteotomy-assisted (model B) and LeFort osteomy I (LFIO)-assisted expansion of the maxillary arch (model C).

**Results:**

Compared with model A, the palatal suture of model B and model C showed a faster stress accumulation rate and shorter fracture time, and the fracture time of model B and model C was almost identical. Compared with model A, we discovered that model B and model C showed greater lateral extension of the maxilla, and the difference was reflected mainly in the lower part of the maxilla, and there was no difference between model B and model C in lateral extension of the maxilla.

**Conclusions:**

Compared with arch expansion using nonsurgical assistance (model A), arch expansion using maxillary lateral wall-osteotomy (model B) or LFIO had a faster rate of stress accumulation, shorter time of fracture of the palatal suture and increased lateral displacement of the maxilla. Compared with arch expansion using LFIO (model C), arch expansion using lateral osteotomy (model B) had a similar duration of palatal suture rupture and lateral maxillary extension. In view of the trauma and serious complications associated with LFIO, maxillary lateral wall-osteotomy could be considered a substitute for LFIO.

## Background

Maxillary transverse deficiency is a common deformity in adolescents and adults. It can cause transverse maxillomandibular discrepancies and posterior crossbite [1–3]. Surgically assisted rapid palatal expansion (SARPE) has become the primary choice for palatal expansion in adults [4–7], but there are controversies regarding the choice of surgical method [8–10]. A LeFort I osteotomy (LFIO) can relieve maxillofacial resistance and concentrate the expansion force [11], and is used commonly for SARPE to accelerate palatal expansion [5, 7, 12].

A LFIO in SARPE has some disadvantages: the side-effects of general anesthesia, risk of fracture, prolonged recovery period and an injury risk to the pterygopalatine segment of the maxillary artery [13–15].

A simplified surgical method has been considered to replace the LFIO. Glassman and colleagues undertook conservative surgery on 16 adults for palatal expansion, and obtained an excellent therapeutic effect [16]. Antilla and coworkers reported the feasibility and long-term stability of lateral osteotomy-assisted maxillary expansion [17]. Recent research has demonstrated the necessity of a paramedian osteotomy and pterygomaxillary separation in partial- and complete-fusion sutures [18]. However, the conclusions of those clinical or basic-research studies were limited by small sample sizes, absence of a control group and lack of biomechanical research.

The finite element analysis (FEA) method was first used to evaluate the mechanical behavior of skeletal parts in 1972. The FEA method is noninvasive, convenient and repeatable. We postulated that a three-dimensional (3D) FEA method could be used to simulate a surgical procedure by weakening the influence and stress of craniofacial-bone resistance and fracture of the palatal raphe. In this way, biomechanical effects could be investigated.

We wished to establish a 3D FEA model of the craniomaxillofacial complex based on fracture mechanics [19]. Then, we aimed to use this modeling method to simulate nonsurgical-assisted, maxillary lateral osteotomy-assisted and LFIO-assisted expansion of the maxillary arch (hereafter termed “arch”). We compared the time required for fracture of the palatal suture, the distribution of craniomaxillofacial stress and the change in displacement of the maxillary complex when arch expansion had been completed. In this way, a theoretical basis for simplified surgery could be provided.

## Materials and methods

### Reconstruction of a 3D FEA mesh model of the craniomaxillofacial complex

This study was conducted using the cone-beam computed tomography (CBCT) data (0.300-mm layer; voxel size, 0.463 × 0.463 × 0.300 mm^3^) of a 21-year-old female volunteer diagnosed with maxillary transverse deficiency and deciduous tooth retention. This study was approved by the Ethics Committee of Xiangya School of Stomatology. CBCT sections were saved as digital imaging and Communications in Medicine images, and then imported to E-3D v16.22 (Hunan, China) for 3D reconstruction. Then, the STL model was exported to Geomagic studio v12 (3D Systems, Rock Hill, SC, USA) in which noise is eliminated from the geometry and the contours are smoothed. The gray value ranged from 100 to 2000 HU, and hard tissue was selected for reconstruction. Then, the Regional Segmentation and Seed Point function were used to detached maxilla and mandibular. The Mask function was used to erase noise from the obtained maxilla. The smooth function was used to generate geometric model of three dimensional surface grid, which was save in STL formation.

The STL model was imported into Geomagic Studio 13.0 software for mesh doctor examination to repair the problematic mesh, then the nails were removed, the holes were filled, the contour lines were edited along the surface of the cranio-maxillofacial complex to generate curved pieces, and the grid was constructed. After fitting the surface, a solid model with high biological simulation was created and stored in STP formation.

**Fig. 1:**
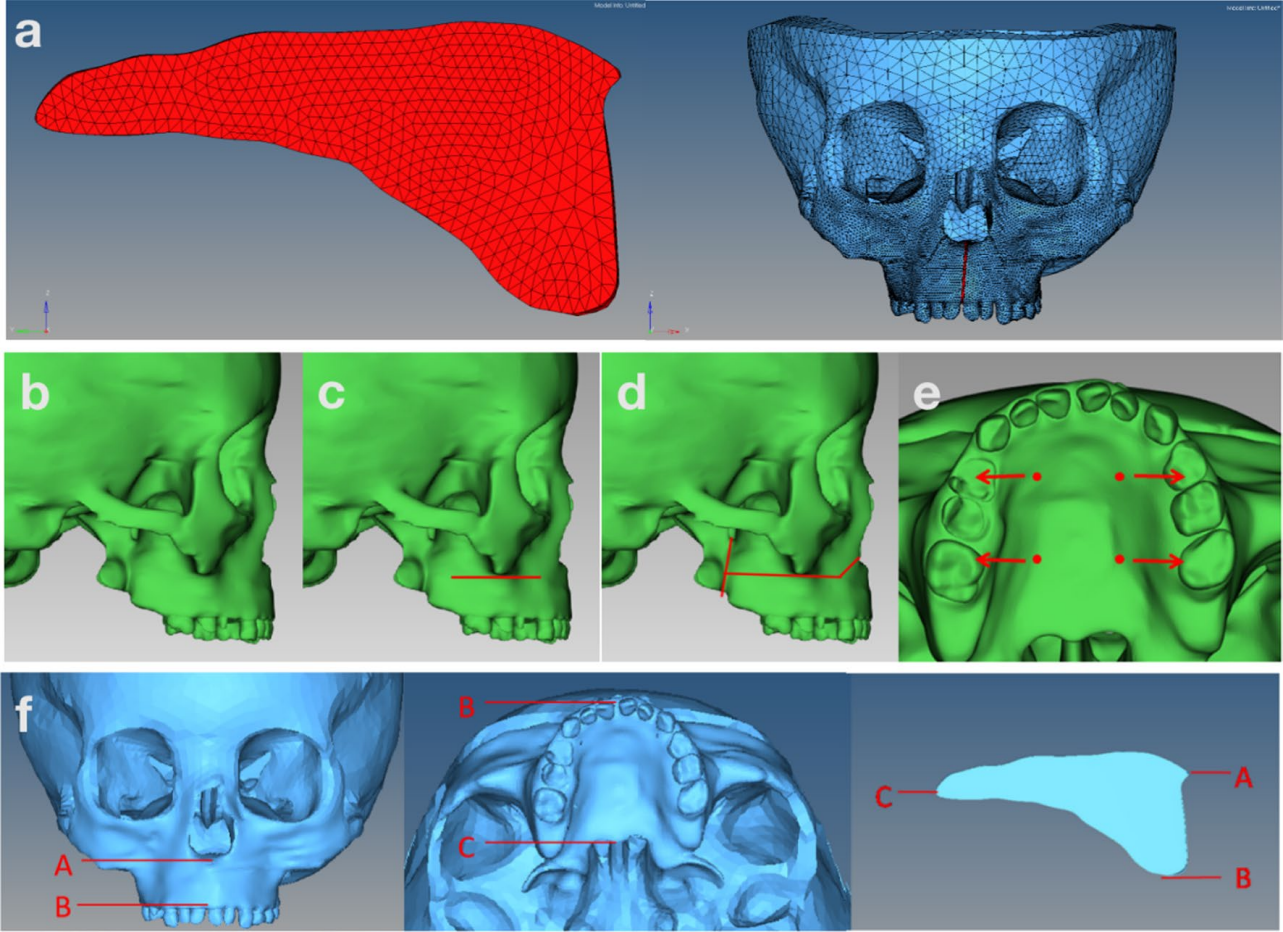
Reconstruction and process of a three-dimensional finite elements analysis of a mesh model. **a** Craniomaxillofacial complex and meshwork of the palatal suture. **b** Nonsurgical-assisted arch expansion. **c** Lateral osteotomy-assisted maxillary palatal expansion. **d** LeFort I osteotomy-assisted maxillary palatal expansion. **e** Loading positions. **f** Red arrows denote measurement positions

The Cranio-maxillofacial complex STP model was imported into CATIA V5 software to construct lateral lateral osteotomy and Lefort1 osteotomy assisted arch expansion model. The surgical scope of Lefort1 included: anterior osteotomy (truncation of nasal and maxillary pillars), lateral osteotomy (truncation of zygomatic and maxillary pillars), and posterior osteotomy (truncation of wing maxillary pillars). In this study, the pedicle extender supported by implant nails was used to simulate arch expansion. In order to facilitate calculation and simplify the model, the palate were divided into four 1.8*5 mm cylindrical geometries to simulate microimplants implanted. Subsequently, the surface FEM were divided into three sizes: 1 mm, 3 mm and 5 mm, based on the rules that the tissue closer to the palatal suture with higher quality FEM structure. Therefore, the diameter for the palatal suture, the maxilla as well as the surrounding adjacent structures is 1 mm, and 5 mm for the parietal and occipital bones, while 3 mm for the rest. Poisson’s ratio and the elastic modulus were set in the FEA to distinguish the bone cortex and cancellous bone. Then, 3D models with different osteotomies were imported into Hypermesh 13.0™ (Altair, Frisco, TX, USA), which was used to create the FEA models. The resulting FEA models (Fig. 1a) comprised an average of 245,516 elements and 45,585 nodes. The palatal cleft contained 1207 nodes and 3302 solid tetrahedral units. This modeling method was used to simulate three methods of palatal expansion: nonsurgical-assisted (model A); lateral osteotomy-assisted (model B); LFIO-assisted (model C). A schematic diagram of surgical incisions is shown in Fig. 1b–d.

### Material parameters, boundary conditions and load setting

The displacement and rotation of the nodes around the foramen magnum in X, Y and Z directions were set as 0. The treatment method of palatal expansion was simulated. Four nodes of area 1.8 mm × 5 mm were selected from the palate to be loaded with a horizontal force that was increased from 0 to 500 N within 140 ms (Fig. 1e).

The material parameters of the tooth, cortical bone, cancellous bone and midline palatine suture were set according to former research (Table 1). The material at the midline palatine suture was set as MAT-Plastic-Kinematic, and the yield strength was 1 MPa. That is, when the stress applied to the material reached the yield strength that we set, its mesh disappeared gradually to simulate crack propagation until the material broke.

**Table 1:** Properties of the materials used in our simulation

	Young's modulus (MPa)	Poisson’s ratio
Cortical bone	13,700	0.30
Cancellous bone	1370	0.30
Tooth	19,890	0.31
Midline palatal suture	15	0.49

The time required for the palate to reach yield strength (T1), crack initiation (T2) and final fracture (T3) were recorded. Three marking points were selected to evaluate the lateral displacement of the maxillary body after palatal expansion. The marking points are shown in Fig. 1f.

### Volunteer patient

**Fig. 2:**
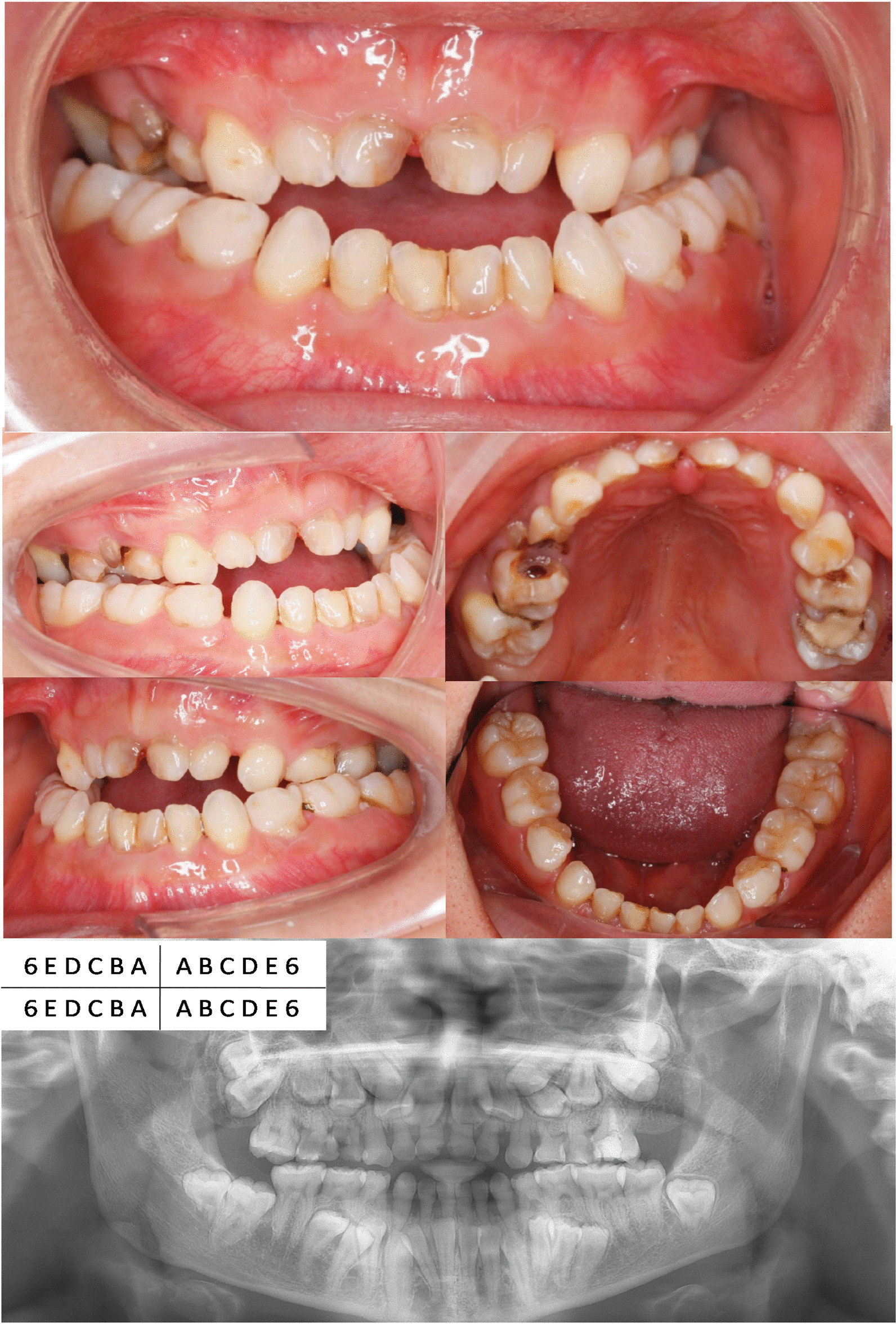
The dental formula of our volunteer

The volunteer was a 21-year-old woman with chief complaints of malocclusion, crossbite and deciduous tooth retention. Intraoral examination revealed deciduous tooth retention, and mixed dentition with class-III malocclusion. The overjet was − 5 mm and overbite was − 3 mm. The dental formula is shown in Fig. 2.

Miniscrew-assisted rapid palatal expansion (MARPE) involves application of a force directly to the maxilla using miniscrews and a skeletal anchorage expander. MARPE was selected for skeletal maxillary expansion. However, the volunteer’s palatal suture was fused. In addition, patients with cleidocranial dysplasia have been reported to have more dense and compact alveolar bone, which indicates that their facial skeleton provides greater resistance to expansion than that of healthy people. Accordingly, MARPE failed to overcome greater resistance or an open, fused midline palatal suture after 30 days of treatment.

**Fig. 3:**
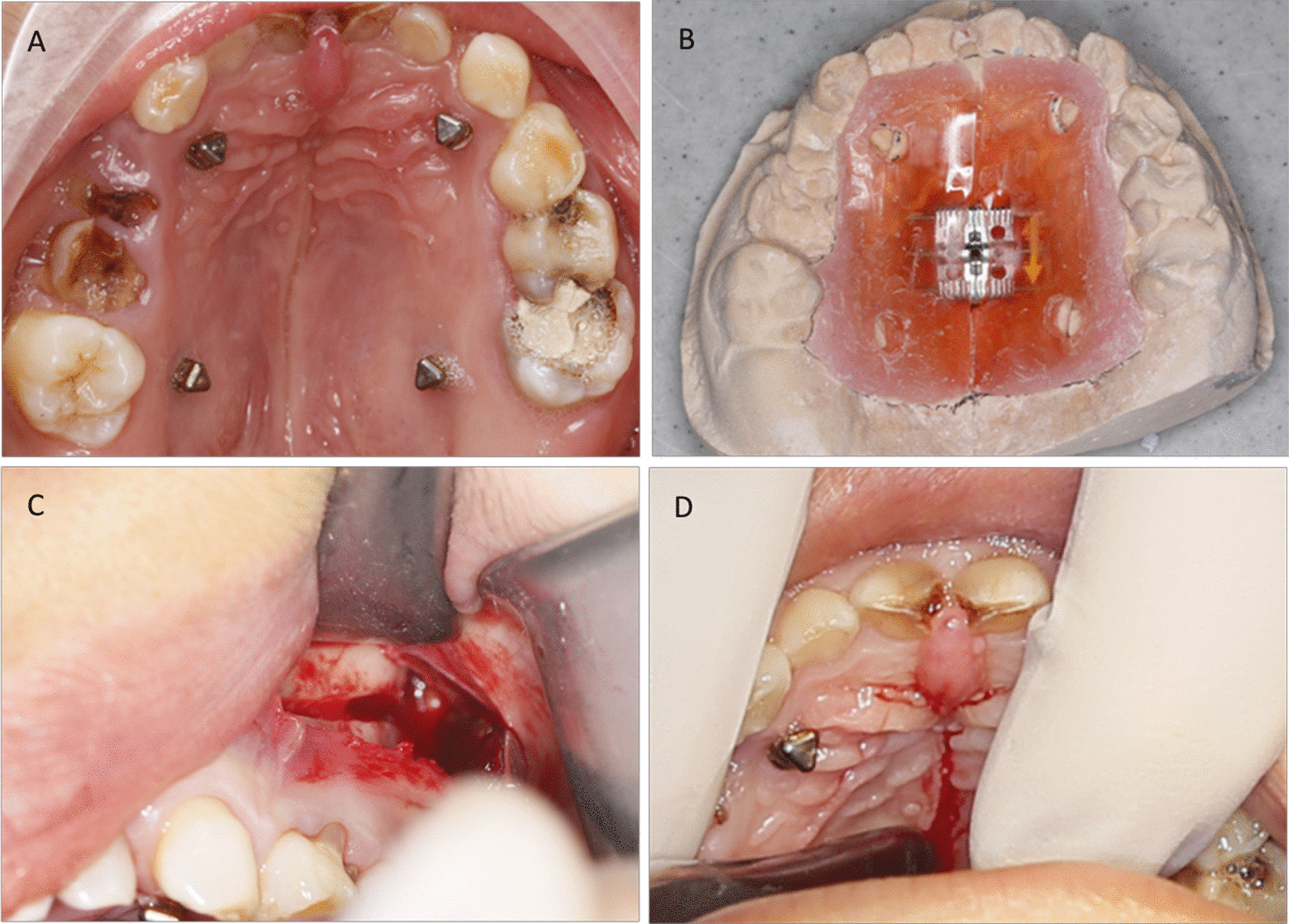
Miniscrew-assisted rapid palatal expansion (MARPE). **a** Fixation of four miniscrews in the palate. **b** Custom-made appliance. **c** Lateral cortiotomy. **d** Mid-palatal cortiotomy

According to FEA results, lateral osteotomy-assisted maxillary palatal expansion was selected, and was completed under local anesthesia in a clinic. Hence, corticotomy-facilitated MARPE was deemed to be the most suitable treatment modality. With regard to the maxilla expander, a custom-made bone-borne device was newly designed to cut costs and reduce invasion. Hence, a new method of corticotomy-facilitated MARPE was developed to resolve maxillary dysplasia while minimizing the side-effects of the procedure. Our treatment plan combined surgery and modified techniques to meet the requirements of our volunteer. The procedure was designed to be more efficacious and less invasive. The patient accepted the option of corticotomy-facilitated MARPE (Fig. 3).

## Results

### Fracture process of the midline palatal suture

The fracture process of the midline palatal suture was similar among the three models. The nonsurgical-assisted palatal expansion model was selected to analyze the process based on fracture mechanics.

**Fig. 4:**
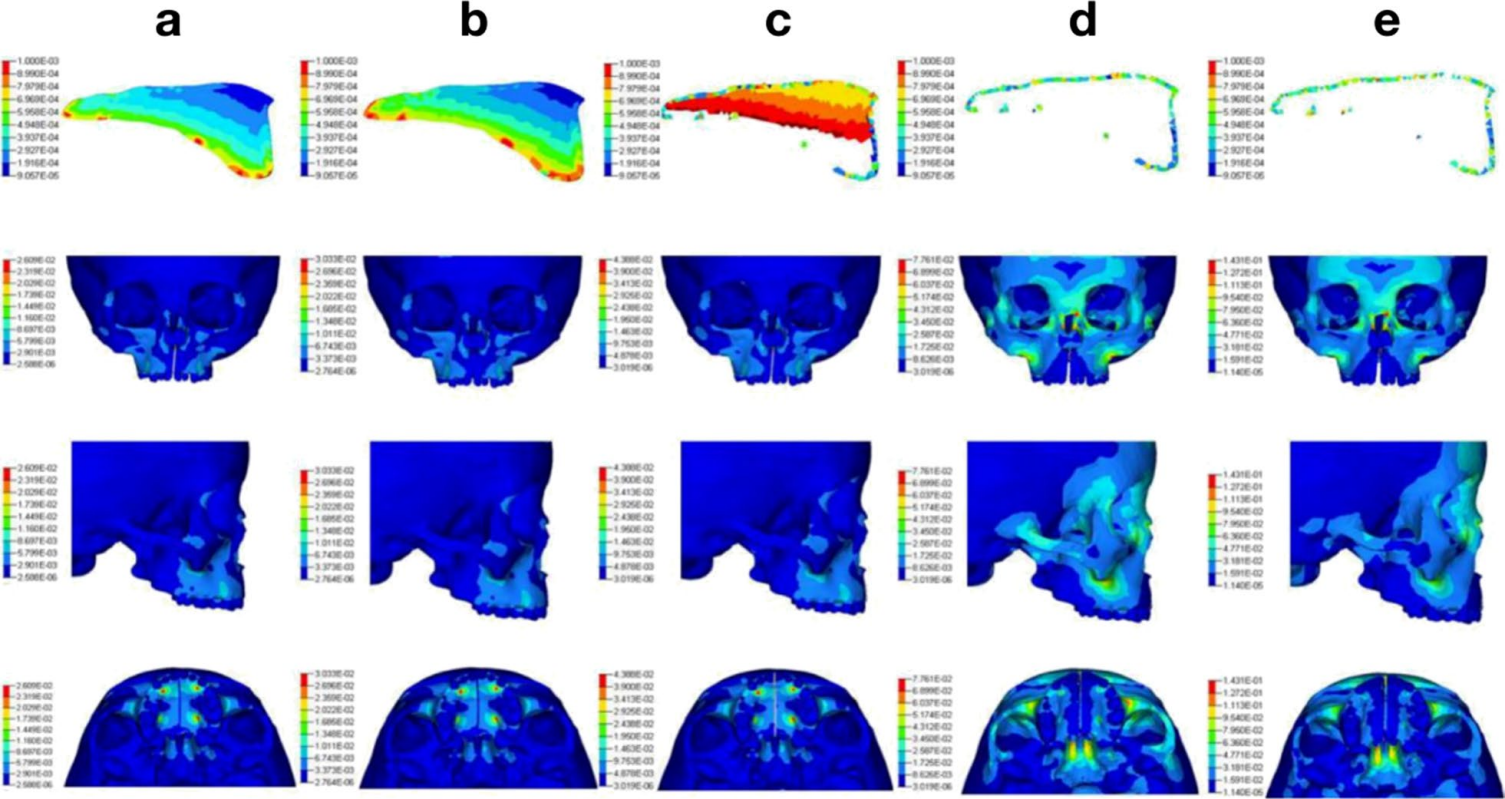
Fracture of the palatal suture and stress distribution of the craniomaxillofacial complex in nonsurgical-assisted arch expansion. **a** The lower mesh before palatal suture reached the yield stress at 52 ms. **b** At 68 ms, the lower mesh began to disappear. **c** The crack continued to propagate upwards and backwards at 80 ms. **d** At 102 ms, the midline palatal suture was completely fractured and the material lost all continuity. **e** At 140 ms, a load was no longer present

From 0 to 52 ms (stress-accumulation stage), the stress on the palate increased gradually until the yield strength was reached (Fig. 4). From 52 to 68 ms, plastic deformation occurred in the midline palatal suture, and the yield stress no longer increased. At 69 ms, an initial crack occurred in the posterior inferior portion because some elements began to erode. From 69 to 102 ms, the initial cracks in the front and lower parts began to expand backward and upward, which represents the process of crack propagation. At 102 ms, the suture was totally fractured. From 102 to 140 ms, displacement of the craniomaxillofacial complex was increased until the end of the expansion.

**Fig. 5:**
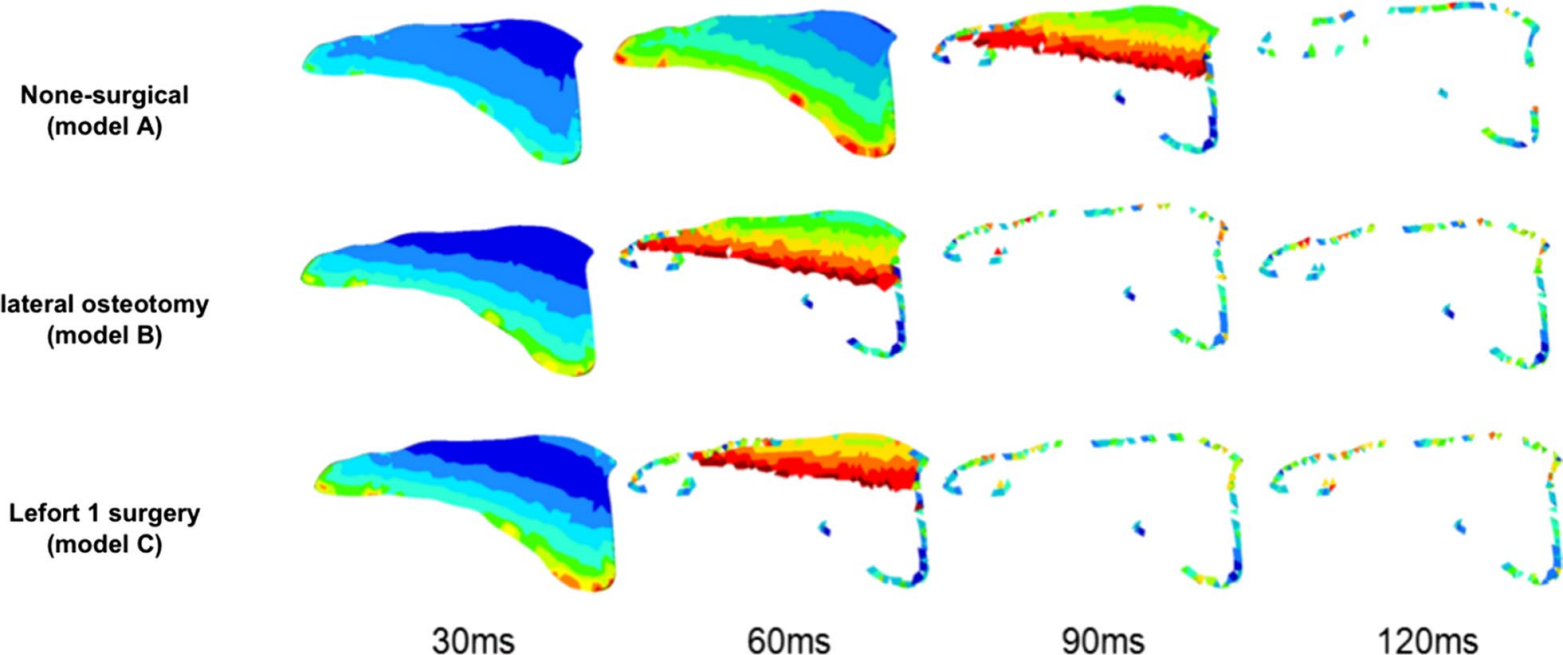
Comparison of the fracture process of the palatal suture between models A, B and C

Stress accumulation and the fracture velocity between different models were compared. The rate at which stress accumulated in the palatal suture was faster in model B and model C than that in model A. At 30 ms, stress in the palatal suture was greater in model B and model C than that in model A (Fig. 5). At 60 ms, crack initiation in model A was absent, whereas crack propagation began in model B and model C. At 90 ms, a crack was observe in model A, whereas the palatal suture was totally fractured in model B and model C.

The time point of the yield strength (T1), crack initiation (T2) and complete fracture (T3) was compared among the three models. As shown in Table 2, the non-surgical group required the longest time for palatal suture fracture. The lateral osteotomy-assisted and LFIO-assisted group had the similar palatal suture fracture rate.

**Table 2:** Timings (T1, T2, T3) in models A, B and C (ms)

	T1	T2	T3
Model A	52	68	102
Model B	36	47	70
Model C	32	37	64

### Craniomaxillofacial stress and strain distribution

**Fig. 6:**
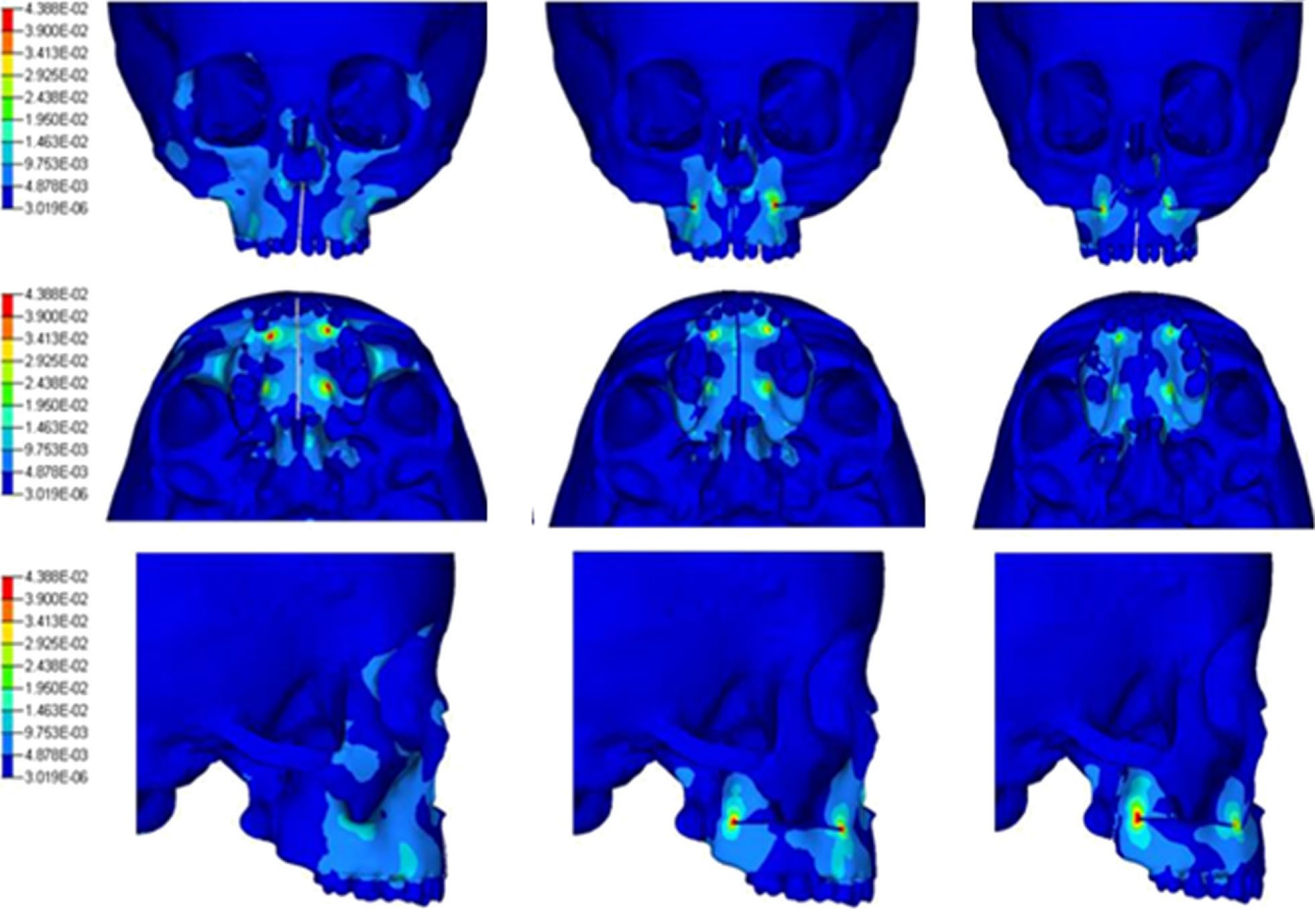
Distribution of craniomaxillofacial stress in models A, B and C before fracture of the middline palatal suture

Before fracture of the palatal suture, for the nonsurgical group, a significant concentration of stress on the zygomatic alveolar ridge was noted. For the lateral osteotomy group and LFIO group, there was a significant stress reduction on the zygomatic crest due to the surgical incision, whereas a concentration of stress occurred on the surgical-incision edge (Fig. 6).

### Lateral displacement of the maxilla

Comparison of lateral displacement of the maxilla is shown in Table 3. From the coronal direction, the maxilla of the three groups presented trapezoidal expansion. The LFIO group had the largest expansion in the anterior and posterior parts.

**Table 3:** Lateral displacement of the measure points (A,B,C) with models A, B and C (mm)

	A	B	C
Model A	2.17	2.93	1.92
Model B	2.26	3.93	2.10
Model C	2.40	4.24	2.24

### Treatment result for our volunteer

**Fig. 7:**
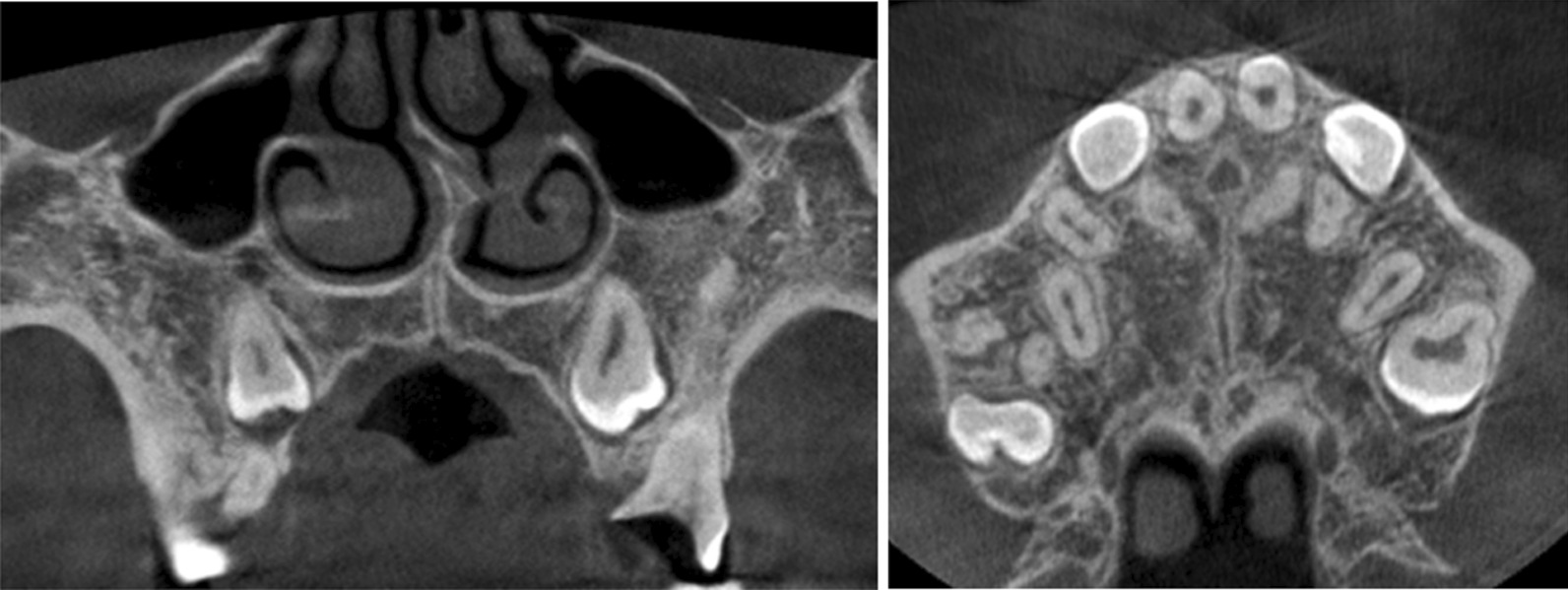
CBCT after MARPE after the first maxillary expansion

A good result was obtained after two expansions. Specifically, after the first expansion, extra space was not observed between the two maxillary deciduous central incisors. Then, CBCT was undertaken to ascertain if the treatment plan was efficacious and realizable. CBCT after first maxillary expansion revealed a crack at the middle–posterior part of the midline palatal suture in transverse section, and the crack extension had reached the nasal septum in the coronal plane (Fig. 7).

**Fig. 8:**
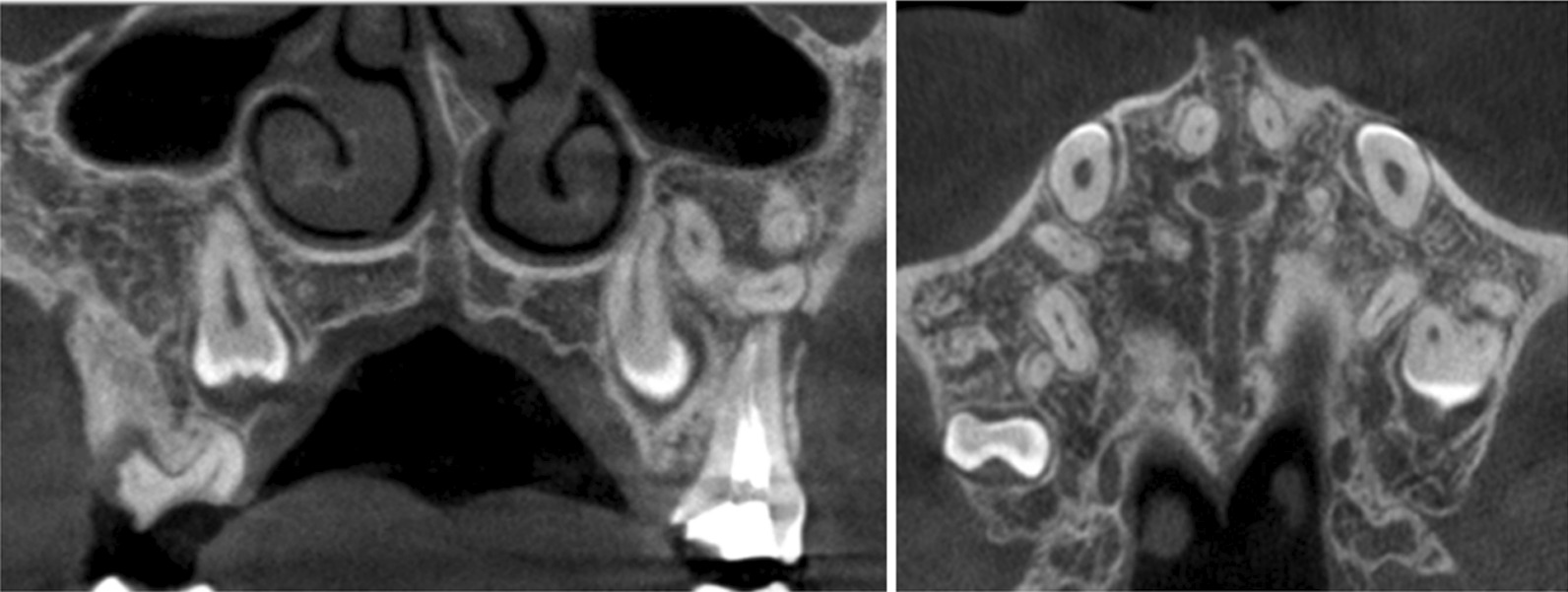
CBCT images 1 month after MARPE

We suspected that stress accumulation was not sufficient to completely expand the midline palatal suture, and that an additional maxilla expander was needed. After the second expansion, the space of the primary central incisor was 4 mm, and posterior crossbite had improved. After 1 month, CBCT was carried out again: the midline palatal suture was completely cracked from front to back and from bottom to top. As a result, the fracture was approximately parallel in the transverse plane, and V-shaped in the front plane (Fig. 8).

## Discussion

Studies have considered the posterior wall of the maxilla to be one of the main resistance areas during arch expansion [5, 13, 20]. To achieve greater arch expansion, the posterior connection of the maxilla must be truncated. However, some clinical studies in recent years have proffered different opinions. In 2014, Sygouros and colleagues collected 20 cases of SARME for retrospective analyses: 10 cases were truncated and the remaining 10 cases were not [21]. They found no significant difference in maxillary dilatation between the two groups, but the group with a untruncated maxillary junction showed more buccal inclination in the posterior tooth segment. The purpose of our research is to evaluate whether osteotomy of the lateral maxillary wall can be superior to the LFIO, considering its advantages in local anesthesia and other complications [22–24].

Figure 4 shows the initiation and propagation of cracks in the region of the palatal fissure after model A had been subjected to a continuous expansion force in the arch. Eventually, macroscopic cracks formed to cause fractures in the palatal suture area, thereby achieving the therapeutic effect of bony expansion. This type of fracture follows the process of crack initiation, crack propagation and material fracture under a continuous load. Also former research [1–3] concluded that no significant difference was found between both surgical techniques with regard to the different parts of the maxilla. With regard to the stress distribution of the three models (Fig. 4), in the nonsurgical group, the stress concentration was in the anterior, lateral and posterior walls of the maxilla. However, the stress in the zygomatic alveolar ridge was relatively high, indicating that the lateral wall of the maxilla may be the most important source of arch-expansion resistance, and that accelerated fracture of the palatal suture in the lateral wall-osteotomy group could confirm this conjecture. Accordingly we hypothesized that osteotomy of lateral may achieve ideal therapeutic effect.

Table 2 shows a direct comparison of the fracture rate of the palatal suture. The time required for the surgical groups to fracture the palatal suture was significantly shorter than that for the nonsurgical group. Hence, a surgical procedure can realize a reduction of expansion resistance and accelerated fracture of the palatal suture [9, 10, 25]. However, the results for model B were almost identical to those of model C, indicating that even a simple incision in the lateral wall of the maxilla could weaken the resistance of the craniomaxillofacial bone and accelerate fracture of the midline palatal suture.

To further analyze the stress distribution in different model, Fig. 6 was performed to reflect the fracture process of the palatal fissure. Under an identical loading condition, the fracture process of the palatal suture in model B and model C developed faster than that in model C. These results suggested that the palatal suture in the surgical groups accumulated a greater expansion force due to a reduction in resistance of craniomaxillofacial bone. This part of result also shown that the sphenoid body had a high concentration of stress. This region has extremely important structures, such as the trigeminal nerve and middle meningeal artery. Lanigan and colleagues reported on skull base (SB) fractures and ruptures of the middle meningeal artery caused by surgically assisted arch expansion [26]. Those complications could be related to the complex structure of the SB and excessive stress accumulation during arch expansion. Using 3D FEA, Holberg and coworkers simulated a large wing of the sphenoid bone moving outwards by 2 mm, and found that the stress on the SB increased significantly [27]. We showed that the stress distribution in the SB remained high even after incision of the pterygomaxillary junction. This phenomenon may have occurred because the maxilla continued to have a connection with the surrounding bones even in SARME using LFIO. For example, the inner wall of the maxilla and outer wall of the nasal cavity have extensive connections, so the stress of arch expansion can be transmitted to the SB. To fully protect important SB structures, a segmental maxillary osteotomy can be considered, whereby the maxilla is disconnected completely from the surrounding bone, allowing the maxilla to move freely.

We hope to not only accelerate fracture of the midline palatal suture through appropriate surgery, but also to increase expansion of the upper jaw. Table 3 shows that lateral displacement in the lower maxilla of the lateral wall-osteotomy group was larger than that of the nonsurgical group, and slightly smaller than that of the LFIO group. Hence, “ideal” maxillary expansion could be achieved even if the lateral wall-osteotomy of the maxilla was straightforward. Anttila and coworkers selected 20 patients (mean age = 31 years) with lateral osteotomy-assisted RME of the arch [17]. After arch expansion, the mean width between canines and molars increased by 4.2–7.1 mm. Two years later, the mean width between canines and molars decreased by 0.5–1.3 mm, thereby achieving a stable and ideal therapeutic effect.

Despite all the advantage, our study still had some limitations. Firstly, FEA cannot perfectly represent the complicated structure of human skull [28]. This limitation also emphasize the necessity of clinical study, which confirms these findings from FEA. Secondly, compared with the tetrahedral element, the hexahedral element can improve the accuracy of calculation to a certain extent. However, the craniofacial anatomic structure is complex, and the tetrahedral mesh has better adaptability to complex geometry. In the future, we can consider replacing the tetrahedral element with the hexahedral element to improve the calculation accuracy. Last but not least, in the application of isotropy and anisotropy in finite elements, although the results simulated by anisotropy are more realistic, medical biomechanics uses isotropy primarily, and relevant reference points are needed. In the future, we can combine micro-CT and isotropy for investigations.

## Conclusions

Our study elicited three main findings. First, compared with arch expansion using nonsurgical assistance (model A), arch expansion using maxillary lateral wall-osteotomy (model B) or LFIO had a faster rate of stress accumulation, shorter time of fracture of the palatal suture and increased lateral displacement of the maxilla. Second, compared with arch expansion using LFIO (model C), arch expansion using lateral osteotomy (model B) had a similar duration of palatal suture rupture and lateral maxillary extension. Third, in view of the trauma and serious complications associated with LFIO, maxillary lateral wall-osteotomy could be considered a substitute for LFIO.

## Data Availability

Not applicable.
